# Ought Members of the Active Medical Staff to Sit upon Hospital Boards?

**Published:** 1890-02-22

**Authors:** 


### Passing Topics.

### OUGHT MEMBERS OF THE ACTIVE MEDICAL STAFF TO SIT UPON HOSPITAL BOARDS?

This chronic question, which has been slumbering awhile, has suddenly taken an acute form at the German Hospital. The committee of management of that institution has hitherto been composed of eighteen members, all "laymen," and the medical staff being dissatisfied with this arrangement, for what definite reason, if any, is not stated, has demanded direct representation, and having failed to persuade the committee to receive their representatives voluntarily, brought forward a motion at the general meeting of the subscribers, the effect of which would be to compel the committee to admit to their body two delegates from the staff. That motion was carried at the meeting by 93 votes to 88, whereupon a poll was demanded on behalf of the committee, and will be taken on the last day of the present month.

No more serious calamity can befall a hospital than that the lay and medical authorities should be involved in public hostilities and happily for the well-being of our institutions, such occasions, owing to mutual respect and forbearance, are comparatively rare. None the less, grave difficulty, generated by the very question now agitating the German Hospital, and capable at any moment of bringing about trouble and even disaster, may underlie the seemingly smooth surface of affairs elsewhere, and it is certain that sooner or later this subject will need to be faced and disposed of.

The practice at different hospitals is entirely dissimilar. While the medical element is in some cases all powerful in regard to management, in others, perhaps, it possesses no adequate means of making known its views upon matters which legitimately concern it. The greatest hospitals—those which circumstances render most independent—have rigorously excluded members of the active staff from a share in the management; at St. George's, where there is an "open board," the members of the staff have no position in relation to management given them in their staff capacity, but they are at liberty to qualify for governorship if they please by paying the required subscription in the ordinary way, and at some of the smaller general hospitals and a great majority of the specials, the members of the staff are in larger or lesser proportions admitted to seats at the committee.

Respecting these three examples it may be observed that in the first group we have the result of experience and deliberate judgment arrived at without distracting influences; in the second there is the somewhat negative result that members of the staff do not sit in their staff capacity, but are not disqualified because of it; and in dealing with the third, we must not forget that the vast proportion of the smaller special hospitals are the creation of medical men who have put themselves into such positions of authority—occasionally of paramount authority—as they are found to occupy.

These considerations tend to depreciate the apparently formidable statistics put forward in support of the resolution, and all reasoning based merely upon a belief in the infallibility of what is seems defective. If it be undesirable that members of the active staff shall sit upon Boards of Management, it is not the less undesirable because they commonly do so. The smaller hospitals mostly are "in pawn" to their medical officers, and they have never had any real choice in the matter. It is not the most frequent, but the most perfect form of government which should be sought after. Can we say that St. Bartholomew's and the London, for example, the two largest of our hospitals—the one endowed, the other unendowed, and both without representation of the staff upon the Board—are less capably managed than the petty institutions at the other end of the scale? Or take the German Hospital itself, no one will gainsay Baron von Schroeder's contention that the institution has prospered under the system of management which has hitherto obtained; neither does it appear unreasonable to argue, as he and the committee do, that active officers, whether medical or lay, honorary or paid, should not be placed in a position which might cause them to sit in judgment on themselves.

The matter is worthy of all serious consideration, and we shall propose to pursue it further. Agricola.

